# Double-Injected Human Stem Cells Enhance Rehabilitation in TBI Mice Via Modulation of Survival and Inflammation

**DOI:** 10.1007/s12035-017-0683-3

**Published:** 2017-07-24

**Authors:** Chul Kim, Ji-Min Park, TaeHo Kong, Seungmin Lee, Ki-Weon Seo, Yuri Choi, Young Sook Song, Jisook Moon

**Affiliations:** 1 Department of Biotechnology, College of Life Science, CHA University, Pangyo-ro 335 beon-gil, Bundang-gu, Seongnam-si, Gyeonggi-do, Seoul, South Korea; 2 General Research Institute, CHA general Hospital, Seoul, South Korea; 3 Present Address: SK Chemicals, Eco-Hub, 332 Pangyo-ro, Bundang-gu, Seongnam-si, Gyeonggi-do 13493 South Korea

**Keywords:** Traumatic brain injury, Human placenta-derived mesenchymal stem cells, Cell transplantation, Double injection, Functional recovery

## Abstract

Traumatic brain injury (TBI), a complicated form of brain damage, is a major cause of mortality in adults. Following mechanical and structural primary insults, a battery of secondary insults, including neurotransmitter-mediated cytotoxicity, dysregulation of calcium and macromolecule homeostasis, and increased oxidative stress, exacerbate brain injury and functional deficits. Although stem cell therapy is considered to be an alternative treatment for brain injuries, such as TBI and stroke, many obstacles remain. In particular, the time window for TBI treatment with either drugs or stem cells and their efficacy is still vague. Human placenta-derived mesenchymal stem cells (hpMSCs) have received extensive attention in stem cell therapy because they can be acquired in large numbers without ethical issues and because of their immune-modulating capacity and effectiveness in several diseases, such as Alzheimer’s disease and stroke. Here, we tested the feasibility of hpMSCs for TBI treatment with an animal model and attempted to identify appropriate time points for cell treatments. Double injections at 4 and 24 h post-injury significantly reduced the infarct size and suppressed astrocyte and microglial activation around the injury. With reduced damage, double-injected mice showed enhanced anti-inflammatory- and TNF-α receptor 2 (TNFR2)-associated survival signals and suppressed pro-inflammatory and oxidative responses. In addition, double-treated TBI mice displayed restored sensory motor functions and reduced neurotoxic Aβ_42_ plaque formation around the damaged areas. In this study, we showed the extended therapeutic potentials of hpMSCs and concluded that treatment within an appropriate time window is critical for TBI recovery.

## Introduction

Traumatic brain injury (TBI) is a leading cause of death and disability in adults. Annually, approximately 1.7 million people experience TBI, 17,000 people die in the USA, and 10 million undergo death and/or hospitalization worldwide. More than half of survivors of severe TBI suffer from functional deficits in cognition, memory, and personality over the years [1–3]. TBI is a complicated pathological process. Primary mechanical insults to the brain tissue, such as external force, hemorrhage, and axonal shearing, initially generate TBI accompanied by necrotic cell death of neurons and glia, blood vessel damage, and diffuse axonal degeneration. The insults then lead to the initiation of complex secondary injuries, including increased pro-inflammatory cytokines and chemokines, cytotoxic neurotransmitter release, disturbed calcium homeostasis, mitochondrial dysfunctions, and increased oxidative stress. Finally, TBI leads to brain damage and functional deficits [2, 4, 5]. Despite extensive investigations on the secondary damage mechanisms, no pharmacological treatments that target a single secondary injury factor have shown favorable results to date [6].

Cell therapies that use a variety of adult mesenchymal stem cells (MSCs) for TBI have been suggested as a promising strategy due to their multiple actions after transplantation, including trans-differentiation, systemic anti-inflammatory effects, and the release of various neurotrophic factors. Bone marrow MSCs reduce pro-inflammatory interleukin 1β (IL-1β), IL-6, IL-17, tumor necrosis factor α (TNF-α), and interferon γ (IFN-γ) and increase anti-inflammatory IL-10, transforming growth factor β1 (TGF β1), and TNF-α-stimulated gene/protein 6 (TSG6) [7, 8]. In addition to the local responses, the transplanted stem cells systemically modulate immune responses through the stimulation of the spleen to produce anti-inflammatory IL-4 and IL-10 and neurotrophic factors, such as neuronal growth factor (NGF), neurotrophic factor 3 (NT3), and brain-derived neurotrophic factor (BDNF) [7, 9].

Although many drug and cell treatments have been used, the critical time points of the treatments are controversial, generating huge variations in the extent of recovery and uncertainty. Treatment between 3 and 24 h post-injury with drugs and/or cells has been known to influence the spectra of recovery in human patients and animal TBI models [10–12]. These unsuccessful and unclear outcomes may come from the following difficulties: most clinical trials have been conducted with drugs that target particular pathological pathways, which may be have limited value in the complicated TBI pathogenesis, and little evidence is available on the treatment time points and their associated influence on TBI progress. The latter information is especially crucial in the development of proper treatment protocols and therapeutic approaches.

Here, using a controlled cortical impact TBI model, we evaluated the therapeutic potential of human placenta-derived MSCs (hpMSCs) for TBI with treatment at different time points. The hpMSCs are reported to have self-renewal and differentiation potentials, strong regenerative and immune-modulating capacities, and efficacy in various disease models, including Alzheimer’s disease and stroke [11, 13–16]. Compared to untreated and single-treated mice at 24 h post-injury, double hpMSC injected mice at 4 and 24 h post-injury displayed (1) more competent suppression of inflammation and oxidative stress, (2) enhanced TNFR-mediated survival responses, (3) reduced infarct size, (4) astrocyte and microglial inactivation at 3 days post-injury, (5) better sensory motor functions up to 5 weeks, and (6) suppression of Aβ_42_ plaque formation up to 10 weeks after injury. These data demonstrate, for the first time to our knowledge, that the hpMSCs and their transplantation at two early time points are crucial for TBI treatment to suppress initial injurious responses as well as Aβ_42_ plaque-mediated damage.

## Materials and Methods

### In Vitro Scratch Model for TBI

To investigate the time lapses in the expression of inflammatory factors, a scratch injury assay, a TBI in vitro model, was conducted with the mouse hippocampal neuronal cell lines, HT22. Briefly, after they were at 80% confluence on a 100-mm dish, HT22 cells were differentiated in fetal bovine serum (FBS)-free DMEM (Gibco) for 24 h. After overnight culture, the HT22 cells were injured by a scalpel and cut 20 times. Total RNA was isolated at 4, 8, 12, and 24 h after damage using an RNeasy Minikit (QIAGEN) according to manufacturer’s instructions [17]. Total RNA was isolated from five samples in a single experiment and the experiment was repeated three times.

### Animals

All experiments were conducted with 2-month-old C57BL/6 male mice from Orient-bio, Inc. (Korea). Mice were housed in standard cages with a 12-h light/dark cycle and had free access to food and water. All animal procedures were approved by the CHA University Institutional Animal Care and Use Committee (IACUC) in accordance with the Guide for the Care and Use of Laboratory Animals. The number of IACUC is 170028.

### Isolation and Culture of hpMSCs

Human term placentas (≥37 gestational weeks), which had normal medical, obstetrical, and surgical histories, were obtained by Caesarean section. All donors provided written, informed consent prior to donation. The collection of the samples and their use for research purposes were approved by the Institutional Review Board (IRB) of CHA General Hospital (Seoul, Korea). Each placenta was dissected carefully. The harvested pieces of tissue were washed several times in phosphate-buffered saline (PBS) and then mechanically minced and digested in Hank’s Balanced Salt solution (HBSS, Gibco) with 2 mg/ml Trypsin (Sigma), 20 μg/ml DNase I (Sigma), 1.2 U/ml Dispase (Gibco), and 1 mg/ml collagenase IV (Sigma) for 30 min at 37 °C. Harvested cells were cultured in a T 25 flask (2 × 10^5^ cells/mm^3^, Nunc) in Alpha-MEM media (Gibco, New York, USA) with 10% fetal bovine serum (FBS, Gibco) and penicillin/streptomycin (Gibco), 25 ng/ml FGF4 (R&D Systems), and 1 μg/ml heparin (Sigma). On the third day, the hpMSCs were trypsinized and counted using a hemacytometer. The injected hpMSCs were cultured in a humidified hypoxic incubator at 37 °C with 5% CO_2_, 92% N_2_, and 3% O_2_ and monitored with an O_2_-sensitive electrode system.

### Experimental Controlled Cortical Impact TBI Mouse Model and hpMSCs Transplantation

A controlled cortical impact (CCI) TBI mouse model was generated with 2-month-old C57BL/6 male mice as described previously [18]. Briefly, mice were anesthetized and placed on a stereotaxic frame. The scalp and epicranial aponeurosis were retracted and a 3.0-mm diameter circular craniotomy was performed with a burr drill, lateral (right side) to the mid-sagittal suture, with the center at the following coordinates: AP = 0, ML = +2.0 from the bregma. In this study, we used a 2.0-mm diameter flat face tip with a slightly rounded edge, a 6-m/s strike velocity, a 2.0-mm strike depth, and a 100-msec contact time. After injury, the impact site was covered with a seprafilm, the skin was sutured, and mice were allowed to recover on a heated pad. Mice were kept at 37 °C via a rectal temperature probe and a feedback temperature controller throughout the duration of the surgery. Damaged mice were randomly divided into different groups, including sham, TP1, and TP2. In the TP1 group, mice were infused at 24 h after injury and the TP2 group mice were at 4 and 24 h after injury. In sham mice, an equal amount of saline was injected. After the CCI procedure, hpMSCs (2 × 10^5^ cells/100 μl PBS) at passage 4 were injected through the tail vein either once (24 h) or twice (4 and 24 h). The same final number of cells was transplanted into the two groups. A total of 96 live mice after injury were randomly divided into each group, sham, TP1, and TP2 and were then subjected to further experiments. Thirty-two mice for TP1 were infused at 24 h after injury and another 32 mice for TP2 were at 4 and 24 h after damage. Eight mice of each group were applied to behavior tests, and total RNA (five mice) and proteins (three mice) were prepared from another eight mice from each group. The rest of mice were applied to histological analysis including MRI scanning.

### Histological Analysis

To confirm disruption of the BBB, 2% Evans blue (Sigma-Aldrich; 4 mL/kg) was administered into the external jugular vein of the animals (*n* = 3 per group) 1 h before sacrifice and damaged mice were anesthetized at 24 h after insult. Animals were perfused with PBS, and the brains were extracted from the skull 1 h after systemic injection of Evans Blue to identify the leakage of the dye. The tissue volume loss induced by the insult was determined in a series of 30-μm-thick brain sections stained with hematoxylin and eosin at 3 days after damage. For the analysis of Aβ_42_ plaques in the CCI brains, the brain was isolated at 10 weeks after injury and serially sectioned, followed by detection with an anti-Aβ_42_-specific antibody (BioLegend, 1:500). For the quantitative analysis of Aβ_42_ plaques size, total plaques from each group were counted, 279 plaques from 4 mice in the sham group, 230 from 5 mice in the TP1 group, and 104 from 3 mice in the TP2 group.

### Measurement of Infarct Size in the Damaged Brain

The brains were isolated at 3 days after injury, and the infarct size was measured with Nissl-stained sections at the same position from nine mice in each group using ImageJ. The percentage of the injured area was calculated as follows.

$$ \left[\left(\mathrm{the}\kern0.5em \mathrm{contralateral}\kern0.5em \mathrm{normal}\kern0.5em \mathrm{hemisphere}\kern0.5em \mathrm{area}\hbox{-} \mathrm{ipsilateral}\kern0.5em \mathrm{area}\right)/\mathrm{twice}\kern0.5em \mathrm{the}\kern0.5em \mathrm{contralateral}\kern0.5em \mathrm{normal}\kern0.5em \mathrm{hemisphere}\kern0.5em \mathrm{area}\right]\times 100. $$

### Behavioral Assays

#### Rotarod Tests

To assess the restoration of sensorimotor function after injury, rotarod tests were performed. A rotarod machine with a 3-cm diameter, automatic timers, and falling sensors (Jeung Do Bio & Plant Co., Ltd.) was used. All mice were trained for three consecutive days on the rotarod each morning. Before each trial, mice were placed on the stationary drum for 3 min. The rotarod was set to start at an initial speed of 4 rpm and to accelerate by 4 rpm every 30 s until reaching 40 rpm. The total running time was 5 min. The tasks were performed three times with 15-min intervals. Mice were injured on the fourth day, and training resumed 3 days after injury. For the test, each trial was terminated when the animal fell off. The latency to falling was recorded, and the average of three trails was analyzed.

#### Balance Tests

A mouse was placed on a round wooden beam with a 0.5-cm diameter at a height of 20 cm. If the mouse maintained balance for 10 s, it passed the test. The score was measured based on the severity: falling = score 0, hanging = score 1, standing = score 2, and walking = score 3.

### Magnetic Resonance Imaging Analysis

Three to four mice from each group were subjected to serial magnetic resonance imaging (MRI) scanning 7 days after injury (6 days after hpMSC infusion). Briefly, the 4.7-T preclinical magnetic resonance imaging (MRI) instrument (BioSpec 47/40, Bruker BioSpin, Ettlingen, Germany) has a 40-cm horizontal bore magnet interfaced to an AVANCE console and is equipped with a 12-cm gradient set (BGA-12) that is capable of providing 720 mT/m with a slew rate of 6000 T/m/s. A birdcage coil with a 70-mm inner diameter was used to transmit and receive the radio frequency (RF) signal. Scout images were acquired in three planes with a gradient-echo sequence to determine the appropriate positioning for the study. A fast spin-echo, rapid acquisition with relaxation enhancement (RARE) sequence was used for the MR images. A sequence with a 256 × 256 matrix was obtained with the following parameters: effective echo time = 90 ms, repetition time = 5000 ms, echo train length = 8, field of view = 2 × 2 cm^2^, and number of averages = 2. Whole-brain coverage was obtained with 1-mm-thick axial slices with no gap. Each animal was anesthetized with isoflurane (5% induction, 1.5% maintenance) in mixed N_2_O and O_2_ (7:3). The rectal temperature was monitored and temperature was maintained with a warm air blower. In order to measure the infarct area, among serially taken images in a row from anterior to posterior part in a single animal, the image at same position closed to the impact region (* marked second one from right) was selected and the inflammatory water content was then measured with ImageJ. The ratio was calculated by the water content on ipsilateral/water content on contralateral regions.

### Quantitative PCR Analysis

Total RNA was extracted from the scratched HT22 cell lines and right hemisphere of the mouse traumatic injury brain (in vivo). RNA was isolated using the RNeasy Minikit (QIAGEN, Germany) according to the manufacturer’s instructions. One microgram of purified total RNA from each sample was converted into cDNA using a Maxime™ RT PreMix kit (Intron, South Korea) and all cDNA samples were stored at −80 °C. To amplify various target genes, 1 μg of cDNA was used with the PCR primers. Quantitative real-time polymerase chain reaction (qRT-PCR) with 1 μg of cDNA was performed using the SYBR-Green reaction kit (Toyobo, Japan) according to the manufacturer’s instructions in a Exicycler™ 96 (Bioneer, Korea). The values for target gene expression were normalized against a housekeeping gene (*GAPDH*) [19]. After normalization with the GAPDH gene, we calculated ΔΔ C_T_ of each genes for an in vitro HT22 scratch assay and in vivo assay with the hemisphere brain from CCI animals. For in vitro assay, the ratio was calculated by (ΔΔ C_T_ of each genes from different time points after injury/ΔΔ C_T_ of each genes from zero time points). The number of samples was five and the experiments were repeated three times. For in vivo assay, the ration was acquired by (ΔΔ C_T_ of each genes from TP1 or TP2/ΔΔ C_T_ of each genes from sham mice). The number of each group of mice was five. The primers used for real-time PCR (Bioneer, Korea) are listed in Table 1.

**Table 1:** The primers used for real-time PCRᅟ

	Forward	Reverse
IL-1β	CCCAAGCAATACCCAAAGAA	GCTTGTGCTCTGCTTGTGAG
IFN-γ	TCAAGTGGCATAGATGTGGAAGAA	TGGCTCTGCAGGATTTTCATG
INF-α	GCTCCAGTGAATTCGGAAAG	GATTAIGGCTCAGGGICCAA
MCP-1	CAAAGAAGCTGTAGITT1TGTCACC	GITCTGATCTCATTTGGITCCG
IL-4	GAACGAGGTCACAGGAGAAG	GAAGCCCTACAGACGAGCT
IL-10	CAGTGGAGCAGGTGAAGAGTG	CAAGGAGTTGTTTCCGTTAGC
SOD-1	GTGCAGGGAACCATCCATT	CATTGCCCAGGTCTCCAAC
SOD-2	CTTACAGATTGCTGCCTGCTC	CTICAGTAACATTCTCCCAGTTG
IL-6	TTCCATCC.AGTTGCCTTCTT	ATTTCCACGATTTCCCAGAG
HIF-lα	TTCAAGCAGCAGGAATTGG	GTAATCCACTCTCATCCATTGAC
VEGF-α	GTGGACATCTTCCAGGAGTA	CTGGCTTTGTTCTGTCTTTC
Nrp-1	AAGAGGTGICATCATTCAGG	AGATCCTGATGAACCTTGTG
FasL	CCAGATCTTCTGGGTAGACA	GATTTGTGTTGTGGTCCTTC
Cas pase 8	ATAACCCAACTCCGAAAAAT	GTGGGATAGGATACAGCAGA
TNFR1	TTCTCCGAGTTTTCCGAACT	ATTGACAACGCTCGTGAATG
TNFR2	CTGAGAGGCCCCAGTATTTT	CAAAAGAATTTCGGTGGGCT
Fas	CTTGCTGGCTCACAGTTAAG	ATGGGCCTCCTTGATATAAT
TRAF2	TTCAGCCTGCAAAAACATCC	GAGAGAATGGATGCACACCT
cIAP-2	ACAGAAGAGCCACGGAAATC	CGCGATACCTTTAAATCGTCC
p50	CTGACCTGAGCCTTCTGGAC	GCAGGCTATTGCTCATCACA
GAPDH	AAGAAGCAGAAAGGCTTCACC	GACGGACACATTGGGGGTAG

### Western Blot Assay

The homogenized ipsilateral brain hemispheres from all groups were lysed in RIPA buffer (Thermo Scientific, USA) with one protease cocktail tablet (Roche, USA) and phosphatase inhibitors II and III (Sigma, USA). Whole lysates were normalized using the BCA assay (Thermo Scientific, USA). Fifty micrograms of each lysate was subjected to 10% SDS-PAGE and Western blot assays. The information on the antibodies used in the experiments includes: anti-total AKT (1:2000), anti-phospho-AKT Thr 308 (1:1500), phospho-AKT Ser473 (1:1500), anti-total ERK1/2 (1:2000), anti-phospho ERK Thr202/Tyr204 (1:1500), anti-Bcl2 (1:1000), anti-Bcl-xL (1:1000), anti-Bax (1:1000) (cell Signaling, USA), and GAPDH (1:3000, Santacruz, USA). Levels of phosphor-AKT and Bcl-xL were calculated with normalized intensity with GAPDH using the ImageJ program.

### Statistics

We performed the statistical analysis with the SPSS software (ver. 22.0, SPSS Inc., Korea). All data were expressed as the mean (standard error (SEM)). The differences among all variables were determined by either ANOVA (post-hoc analysis: LSD) or two-way ANOVA (Mixed model, LSD) test. For the quantitative analysis of Aβ_42_ plaques size, all counted plaque size was transformed into a value of common logarithms and subjected to the Mann-Whitney test. The average plaque size in the TP2 group was analyzed by one-way ANOVA post hoc: Tukey HSD) compared to the sham group or the TP1 group.

## Results

### Analysis of Cellular Behaviors and Time Courses of Changes in Cellular Responses in an In Vitro TBI Model

**Fig. 1:**
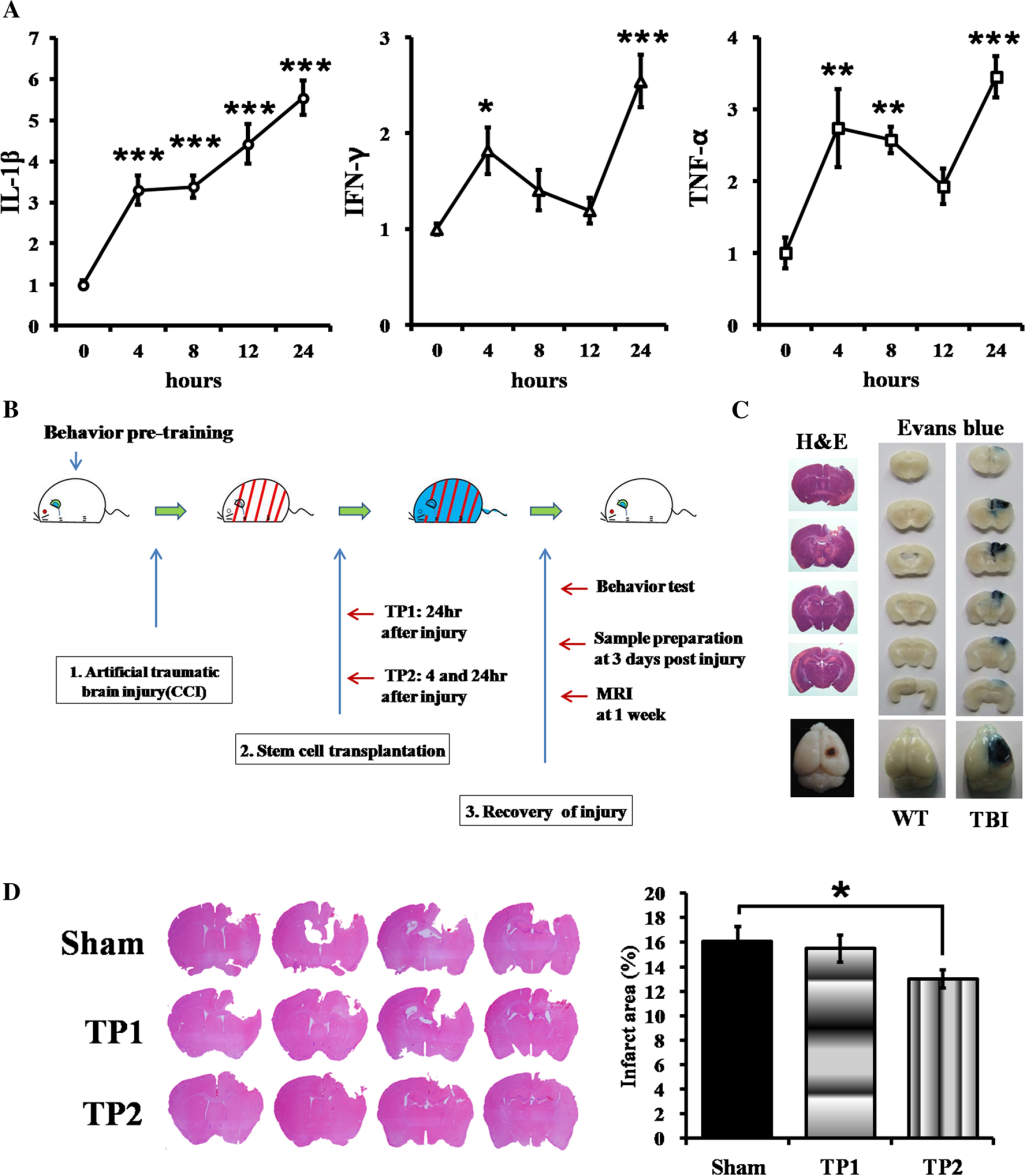
An in vitro model for TBI and a schematic diagram of an in vivo experiment procedure with a controlled cortical impact (CCI) TBI model. **a** In an in vitro scratch TBI model, the damaged HT22 cells induced pro-inflammatory cytokine genes, IL-1β, IFN-γ, and TNF-α at two different time points, 4 and 8–12 h after injury. The quantitative expression of each gene was normalized with GAPDH expression; *p < 0.05, **p < 0.01, ***p < 0.005 (all *p* values are upon S.E.M unless mentioned). b Schematic diagram of an experiment procedure. The pre-trained 2-month-old C57BL/6 male mice were subjected to a CCI. The hpMSCs (2 × 10^5^ cells/100 μl in saline) were then intravenously injected either once at 24 h for the TP1 or twice at 4 and 24 h for TP2 after injury. The same volume of saline was injected into the sham mice. **c** In order to confirm a CCI mouse model, the whole brains were isolated at 24 h after injury and were applied coronal section followed by H&E staining. The CCI brain showed contusion on the cortical region. The damage in the blood-brain barrier (BBB) was displayed by Evans blue injection at 24 h post-injury. The CCI brain isolated 1 h after dye injection displayed infiltration of Evans blue but the wild-type brain did not. The representative figures among the three CCI animals were presented. **d** The serially sectioned brains from sham, TP1, and TP2 mice were Nissl-stained at 3 days post-injury and measured their injury size. TP2 mice displayed significantly smaller lesions compared to sham and TP1 mice. Nine samples from each group were analyzed, *p < 0.05

To determine the time points for stem cell treatment in a TBI animal model, we first investigated the expression patterns of inflammation-related genes, including IL-1β, IFN-γ, and TNF-α, in the injured mouse hippocampal HT22 cells. After injury by a scalpel 20 times, the cells expressed all three cytokines in a step-wise manner: IL-1β was significantly induced at 4 h followed by a further increase in its expression at 8 h after injury, and expression of both IFN-γ and TNF-α was enhanced at the first 4 h and rebounded between 12 and 24 h post-injury (Fig. 1a). These results indicated that HT22 cells displayed two temporal responses, immediate early and late cytokine responses against the injury, and that treatment at the intermittent time may modulate inflammatory responses and the therapeutic efficacy in TBI.

### Generation of a CCI Model of TBI

Consistent with the distinct temporal responses in the scratched HT22 cells, mice with the cortical injury exhibited early peak inflammatory responses within 5 h and then either suppressed or maintained them depending on the factors, suggesting that early treatment may produce better outcomes [20]. To explore the feasibility and time window of TBI treatment with hpMSCs, we generated and injected the hpMSCs into an artificial CCI TBI model as described in Fig. 1b. First, the brain damage in the CCI model was confirmed at 24 h post-injury with hematoxylin and eosin (H&E) and Evans blue staining (Fig. 1c). The serially sectioned brains displayed a lesion cavity at the cortical region in the diencephalon and destruction of the blood-brain barrier (BBB) at the impact area. Based on the two temporal responses in the damaged HT cells in our study and in the published results, we hypothesized that the double treatment at the early and late time points may more efficiently ameliorate the affliction. To evaluate the efficacy of double treatment in TBI, we injected the hpMSCs into the CCI animals at either 24 h (TP1) or at 4 and 24 h (TP2) after injury and measured the infarct sizes of Nissl-stained brains at 3 days post-injury (Fig. 1d). The brains from TP2 mice exhibited significantly reduced infarct sizes compared to the brains from untreated mice (sham, *p <* 0.05). At this time, sham and TP1 mice showed a similar extent of damage.

### The Double Treatment of hpMSCs Significantly Suppressed Pro-inflammatory Genes and Enhanced Anti-inflammatory, Antioxidant, and Angiogenesis-Related Genes

**Fig. 2:**
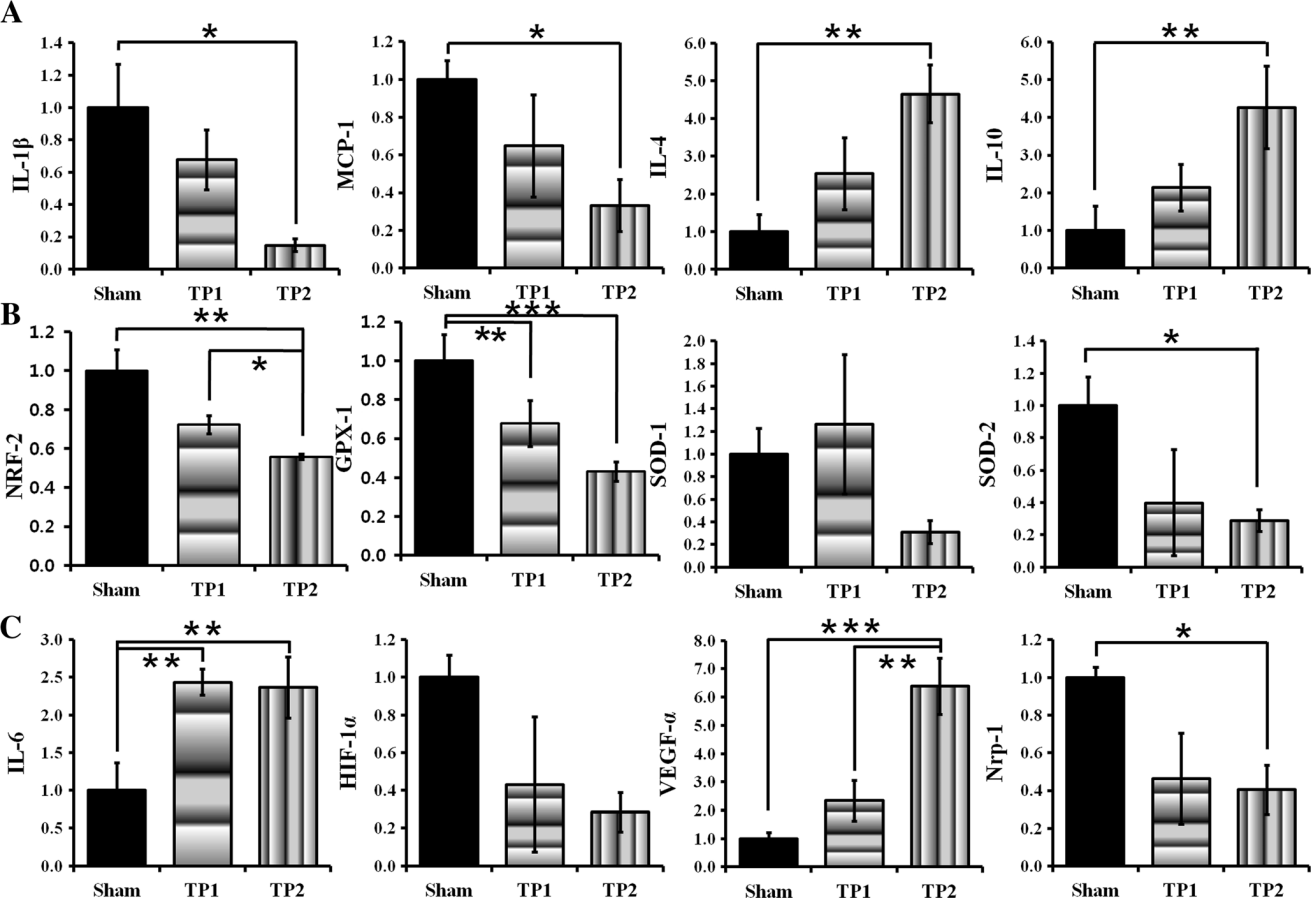
The double injection suppressed pro-inflammatory and oxidative stress-related genes and induced anti-inflammatory and angiogenesis-related genes at 3 days post-injury. **a** Compared to shams, TP2 mice suppressed pro-inflammatory IL-1β, and MCP-1, and induced anti-inflammatory IL-4 and IL-10 genes. **b** As an indicator of oxidative stress, the expression of NRF2 was evaluated among the groups. Compared to sham and TP1, significantly lower level of NRF2 was expressed in TP2 mice. Gpx1, another indicator for ROS and antioxidant gene, also remarkably suppressed in TP2 as well as TP1. Among SODs, key antioxidant genes as well as oxidative stress markers, expression of SOD2, not SOD1, was significantly suppressed by 30% in TP2 compared to the shams. **c** The double injection induced pro-angiogenic genes, VEGF-α and IL-6, and suppressed Nrp-1 compared to the shams. The expression of IL-6 in TP1 was upregulated as much as in TP2. HIF1α expression declined in TP1 and TP2 without significance. Five mice from each group were analyzed, *p < 0.05, **p < 0.01, ***p < 0.005

Because the double treatment mitigated the damage more efficiently, we monitored the expression of inflammatory genes that affect the extent of injury 3 days after the injury. Compared to sham mice, expression of pro-inflammatory factors, such as IL-1β and monocyte chemoattractant protein-1 (MCP-1), was suppressed (*p <* 0.05), but expression of anti-inflammatory molecules, such as IL-4 and IL-10, increased in TP2 mice with significance (*p <* 0.01), but not in TP1 mice (Fig. 2a). These results indicate that the double injection of stem cells is more effective in the modulation of immune responses and elicits the favorable outcomes in TBI recovery.

TBI patients were reported to experience mitochondrial dysfunction-mediated oxidative stress and enhanced inflammation followed by blood vessel damage [5]. Because the double injections strongly modulated inflammatory responses, we investigated whether the transplanted hpMSCs are able to regulate oxidative stress signals and angiogenesis. In order to evaluate oxidative stress in the damaged and the treated brains, we measured expression of nuclear factor E2-related factor 2 (NRF2), an indicator of ROS stress, and other antioxidant genes that are known to increased under oxidative stress such as glutathione peroxidase 1 (Gpx1), superoxide dismutase 1 (SOD1), and mitochondrial SOD2 [21, 22]. Compared to sham mice, expression of NRF2, SOD2, and Gpx1 (*p* < 0.01, 0.05, and 0.001 respectively), but not cellular SOD1, were significantly only suppressed in TP2 mice, indicating that the double treatment of hpMSCs suppresses ROS generation (Fig. 2b). In addition, TP2 mice exhibited strongly enhanced pro-angiogenesis genes, such as IL-6 (*p* < 0.01) and vascular endothelial growth factor (VEGF) (*p* < 0.005), and suppressed neuropilin-1 (NRP-1), a membrane-bound VEGF coreceptor (*p* < 0.05) (Fig. 2c). VEGF expression in TP2 mice was significantly enhanced compared to TP1 mice (*p* < 0.01). Interestingly, expression of IL-6, which is known to induce VEGF and suppress NRP-1 expression, was upregulated in both TP1 and TP2 mice. These data suggest that the double-injected hpMSCs can modulate multiple pathways that affect CCI damage and, as a result, can enhance the restoration potentials in TBI mice.

### The Double-Injected hpMSCs Induced Expression of Genes Associated with TNF-α/Fas Ligand-Mediated Survival Signaling

**Fig. 3:**
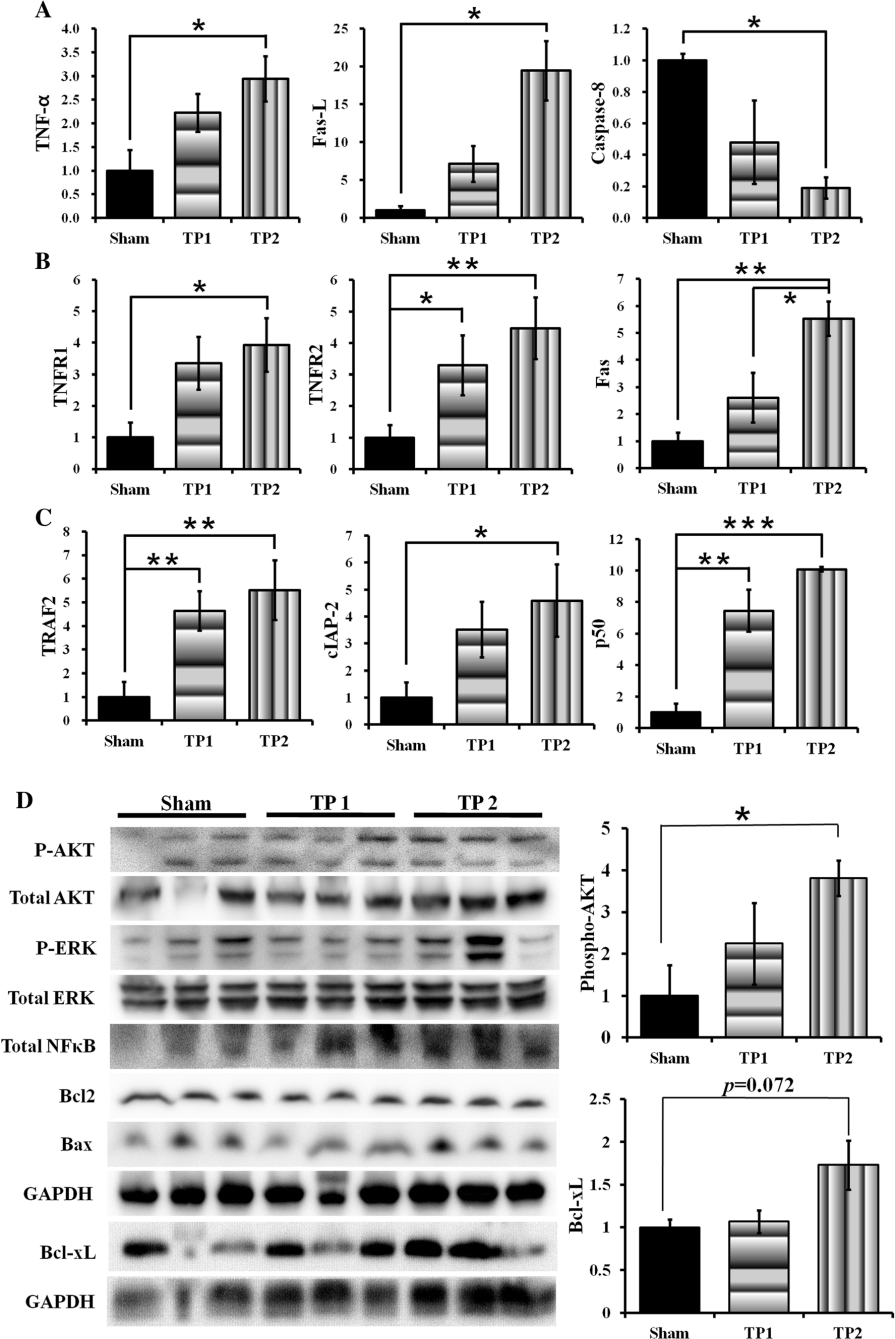
TP2 mice displayed upregulation of TNFR/Fas mediated- and AKT- survival signals at 3 days after injury. **a** Expression of TNF-α and FasL was induced albeit caspase-8 expression was suppressed in TP2 significantly compared to shams. **b** TP2 mice showed increased expression of survival-inducing TNFR2 as well as death-inducing TNFR1 and Fas. Only TNFR2 was significantly induced in TP1. **c** Expressions of TRAF2, cIAP2, and p50 were significantly induced in TP1 and TP2 except for cIAP2. Six to eight mice from each group were analyzed, *p < 0.05, **p < 0.01, ***p < 0.005. Five mice from each group were analyzed **d** Levels of total and phospho-AKT, total ERK, not phospho ERK, and total NFκB were higher in TP2 compared to shams. Significantly higher level of phospho-AKT was observed in TP2 compared to sham (*p < 0.05), and BCL-xL was marginally induced in TP2 compared to the sham (*p* = 0.072609). Three samples from each group were measured

The mechanical damage that mediates necrotic cell death is the primary cause in TBI pathogenesis. Therefore, we investigated whether the hpMSCs modulate the necrotic death signals at 3 days post-injury. Unexpectedly, although expression of TNF-α and Fas ligand (FasL) increased (*p* < 0.05), expression of caspase 8 (*p* < 0.05) was suppressed in TP2 mice (Fig. 3a). Because the Fas/TNF-α receptor (TNFR) complex is able to induce not only cell death but also survival signals in brains through NFκB [23], we investigated the expression of components of TNFR-mediated survival signals, including TNFR2, TNFR-associated factor 2 (TRAF2), a cellular inhibitor of apoptosis protein 2 (cIAP2), and p50 NFκB. TP2 mice exhibited an increased level of TNFR1 (*p* < 0.05) and Fas (*p* < 0.01), and TP1 and TP2 mice both displayed a significant induction of TNFR2, TRAF2, and p50 NFκ-B (*p* < 0.01). cIAP2 was induced in TP2 only (*p* < 0.05) (Fig. 3b, c). These results suggest that the injected hpMSCs not only modulated immune responses but also activated TNFR-mediated survival signals to nullify adverse effects in CCI animals.

### The Double Injection of hpMSCs Activated/Maintained Survival Signals at the Early Stage of TBI Pathogenesis

Next, we investigated whether the cell transplantation altered early survival signals, including AKT, ERK, and Bcl2 family proteins, in the ipsilateral brain region 3 days after injury (Fig. 3d). Compared to sham mice, phospho-AKT and total NFκ-B increased in TP2 mice, but the ERK levels were variable among all groups. In addition to significantly enhanced AKT signals (*p* < 0.05), a trend toward elevation of Bcl-xL (*p* = 0.072) was detected, but the levels of Bcl2 and Bax were unchanged. The differential levels of phospho-AKT and Bcl-xL indicate that the double injections activate or maintain survival signals from the early time point and ameliorate brain damage.

### The Double Injection of hpMSCs Suppressed the Activation of Astrocytes and Microglia in CCI Mice at the Early Time Point After Injury

**Fig. 4:**
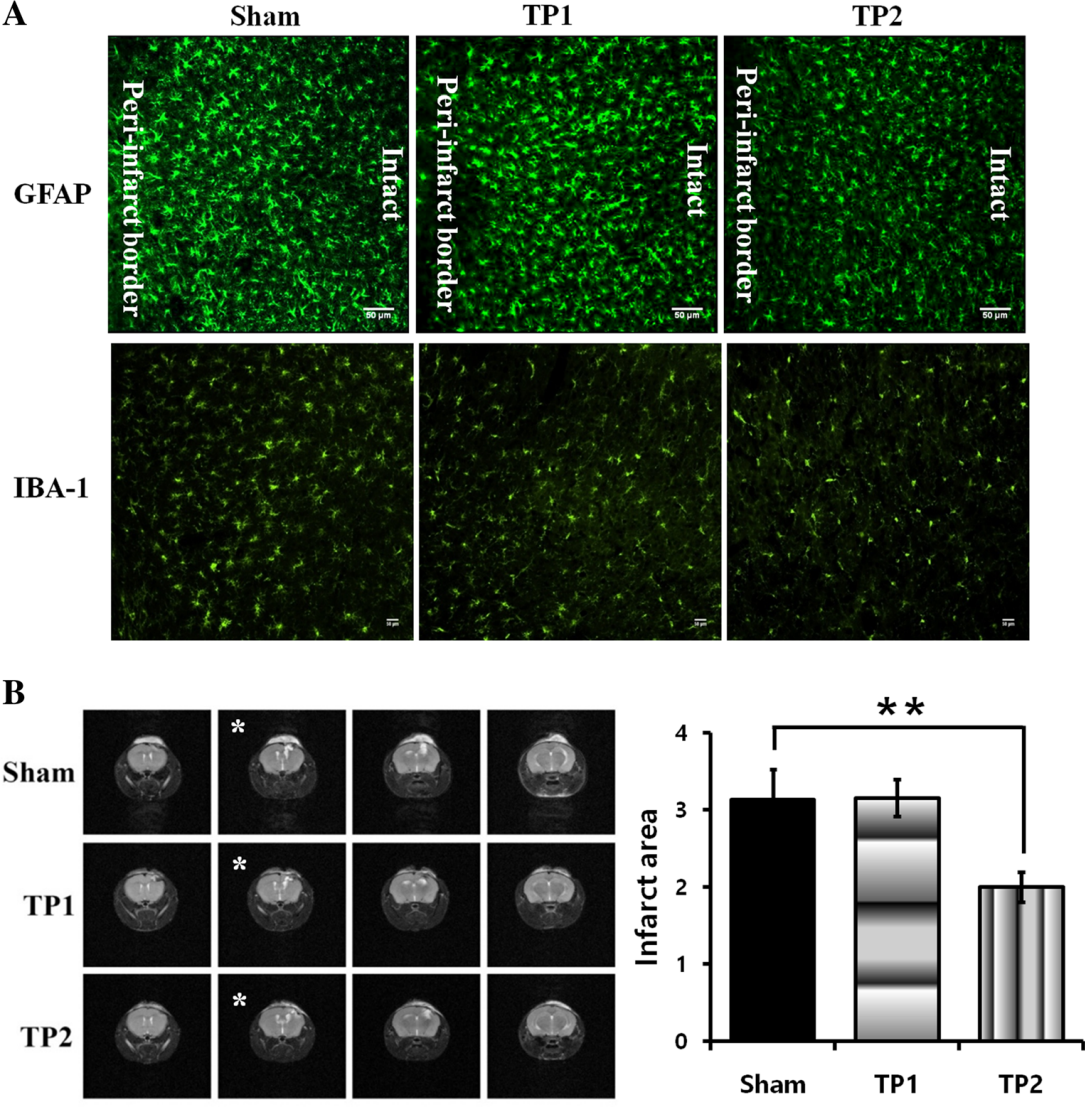
The double-injected hpMSCs suppressed activation of brain innate immune cells at 3 days and reduced immune responses at 7 days after injury. **a** The double injection inhibited activation of astrocytes near the peri-infarct area as well as suppression of microglial activation around the injury sites at 3 days post-injury. The representative figures were presented among 3–4 mice of each group. The *scale bars* represent =50 μm. **b** MRI analysis with the serial planes around lesion areas. Inflammation and/or edema were measured at the closed region to the impact site (the *asterisk*-marked second pictures from right). Compared to the shams, TP2 exhibited reduced white bulb at 7 days after injury, suggesting that the double injection suppressed inflammatory responses and/or edema. Three to four mice from each group were analyzed, *p < 0.05

Because the hpMSCs exhibited modulation of immune responses in the CCI animals, the status of astrocytes and microglial cells, the resident immune cells in the brain, at 3 days post-injury was probed by staining with antibodies against glial fibrillary acidic protein (GFAP) for astrocytes and ionized calcium binding adaptor molecule 1 (IBA-1) for activated microglia. Compared to sham and TP1, the less activated astrocytes were observed near a peri-infarct border and their overall intensity was reduced from the damage to the intact area. Along with suppression of astrocytic activation, much lower intensity of IBA-1 positive microglial cells were detected on the brains from TP2 as well, indicating that double injection is more efficient in the hpMSC-mediated immune modulation in the injured brains (Fig. 4a). However, a clear glial scar was undetectable among all groups of animals. The status of inflammation and/or edema and characteristics of CCI pathology can be detected as white lumps around the impacted cortical region in magnetic resonance imaging (MRI). The serially scanned MRI results at 7 days post-injury showed that the TP2 brains contained remarkably smaller white blobs compared to the sham and TP1 brains, indicating reduced inflammation (*p* < 0.01) (Fig. 4b). These results suggest that the double-injected hpMSCs more efficiently suppressed early inflammatory responses.

### Double-Treated Mice Showed Accelerated Rehabilitation of Sensorimotor Functions Compared to Untreated and TP1 Mice

**Fig. 5:**
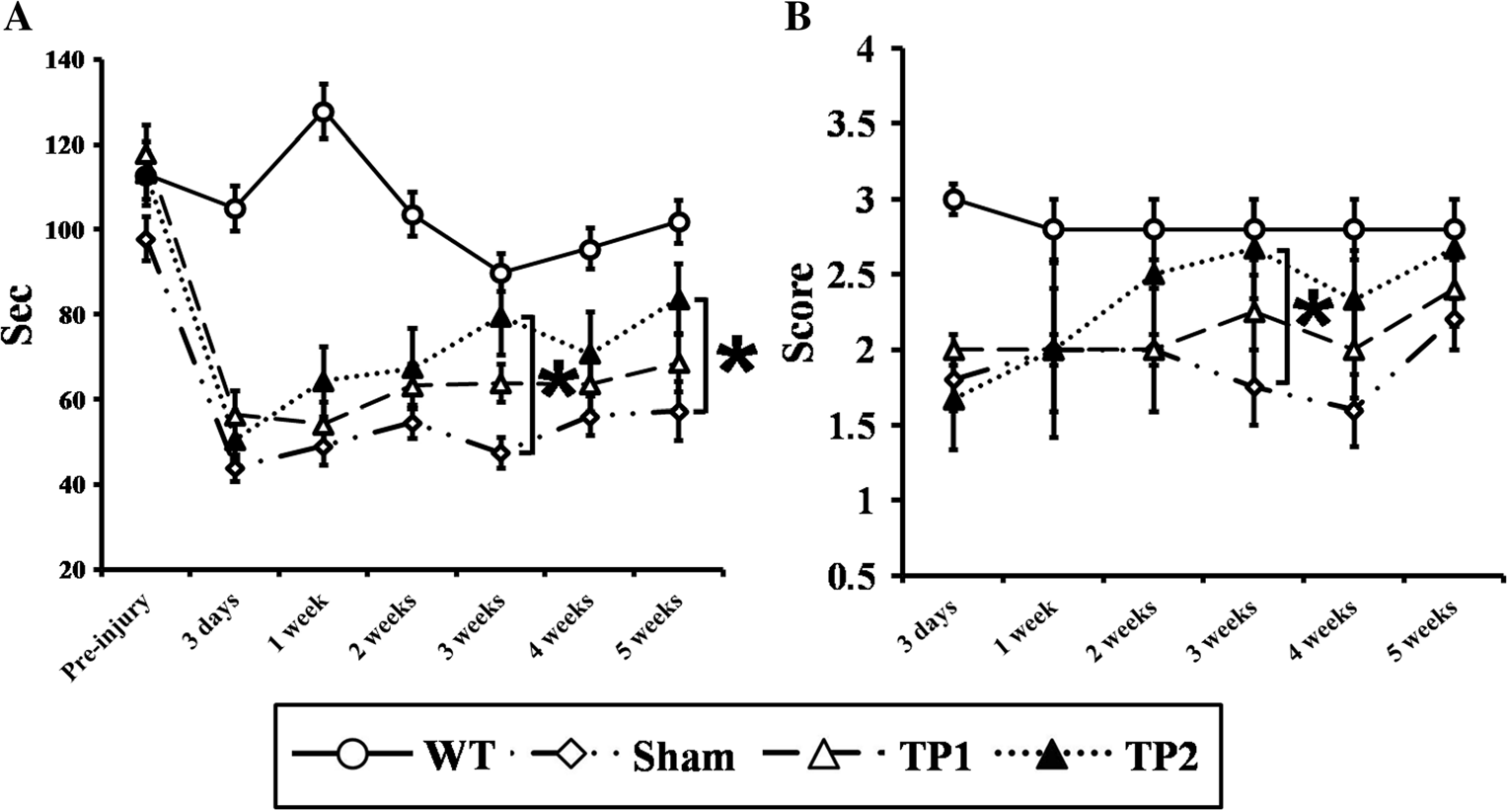
The double hpMSCs treatment improved sensorimotor recovery of the CCI mice. **a** In the rotarod assay, TP2, not TP1, exhibited significantly improved performance from 3 weeks compared to the sham. Eight mice of each group were subjected to the experiments. **b** TP2 showed continued restoration in balance up to 3 weeks after injury compared to the sham. Although TP1 and sham carried out the test as good as TP2 at 5 weeks post-injury, their overall recovery progress was retarded, *p < 0.05

So far, we observed that the double transplantation of hpMSCs into CCI animals enhanced early survival responses and suppressed inflammatory responses. These results prompted us to examine whether the double treatment leads to more favorable functional outcomes in sensorimotor-related behavioral assays, including the rotarod and balance tests. The rotarod test showed that the CCI animals exhibited motor deficits at 3 days post-injury regardless of hpMSCs injection. Both sham and TP1 mice showed sustained failure or slight improvements after 5 weeks, but TP2 group animals displayed improved performance from 1 week and were significantly recovered by 3 weeks after the injury (*p* < 0.05) (Fig. 5a). The superiority of double injections in functional restoration was detected in the balance test as well: TP2 group mice showed continued enhancement from 3 days post-injury and reached a similar level to wild-type control mice (*p* < 0.05) (Fig. 5b). Both results indicate that the double transplantation not only modulates the early cellular responses but also enhances long-term functional rehabilitation compared to other groups.

### TP1 and TP2 Suppressed Astrocytic and Microglial Activation, but Only TP2 Showed the Reduced Accumulation of Aβ_42_ in the Brains of CCI Animals at 10 weeks Post-injury

**Fig. 6:**
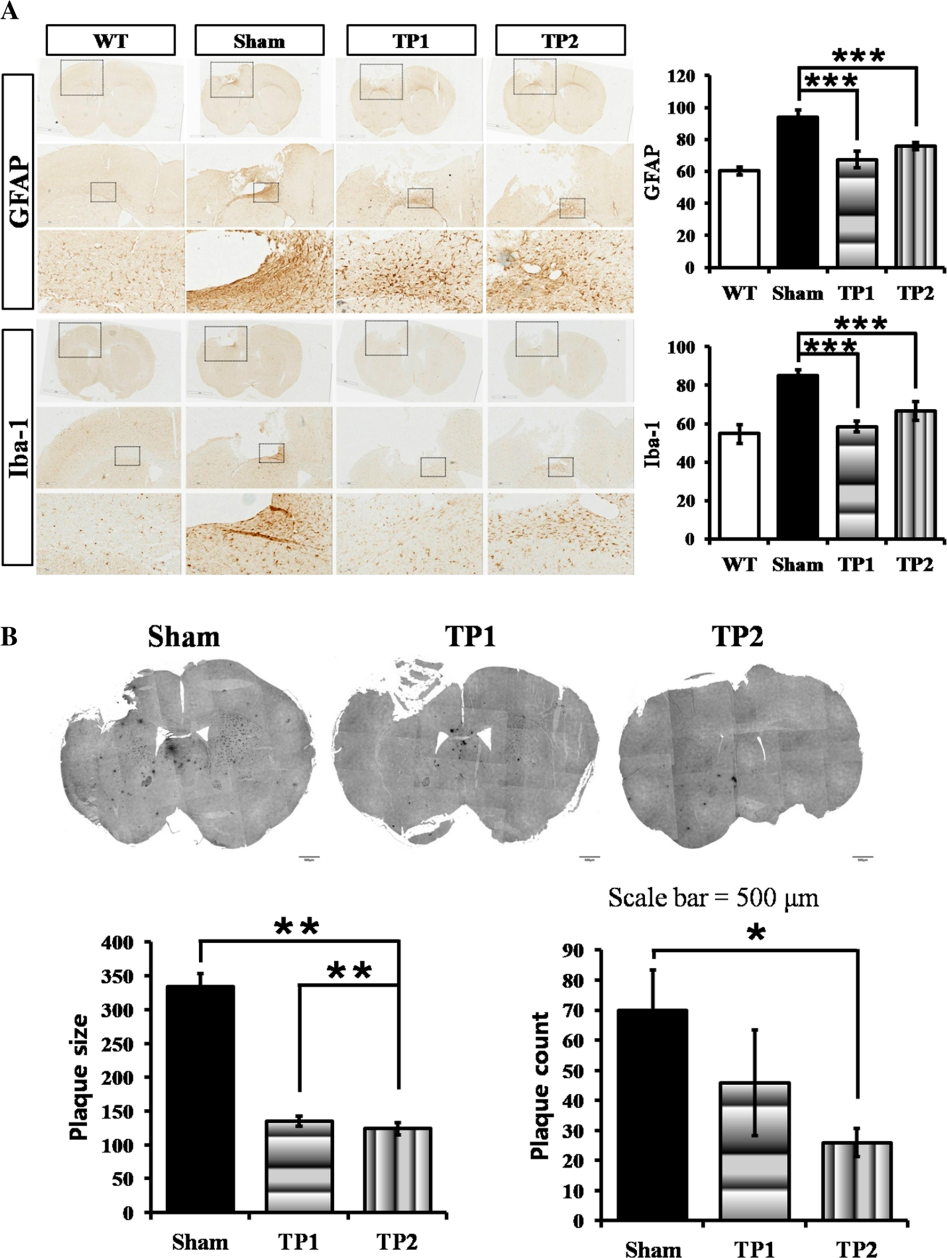
The hpMSC transplantation suppressed astrocyte/microglia activation and formation of neurotoxic Aβ_42_ plaques in CCI mice at 10 weeks post-injury. **a** The activated astrocytes and Iba-1 positive microglias on the brains from both TP1 and TP2 were observed at less extent compared to the shams at 10 weeks post-injury, indicating the early influence by cell transplantation lasted for long periods. The number of each group is three. The *scale bar* represent 4× = 500 μm and 20× = 100 μm; ***p < 0.005. **b** The sizes of Aβ_42_ plaques near the injury site in both TP1 and TP2 were observed significantly smaller, 50% reduction, than that in the sham mice at 10 weeks after injury. Furthermore, compared the sham, the average number of plaques was significantly reduced, less than half, in TP2, not TP1. The number of analyzed sham, TP1, and TP2 was 4, 5, and 3 respectively. Total number of plaques was 279 from sham, 230 from TP1, and 104 from TP2 mice, **p < 0.01, *p < 0.05

Many lines of evidence demonstrate that TBI survivors experienced cognitive deficits and that TBI animal models recapitulate AD pathological phenotypes, such as increased astrocytes, activated microglia, Aβs levels in the cerebrospinal fluid (CSF) and brain, and formation of Aβs plaques [24–26]. We already reported that hpMSC transplantation into AD mice improved spatial learning and suppressed Aβs plaque formation [16]. Therefore, we tested whether the injected hpMSCs influence AD-related phenotypes in the CCI animals at 10 weeks after injury. Compared to sham mice, both TP1 and TP2 mice displayed less GFAP-positive astrocytes and Iba-1-positive microglia in the subcortical region (*p* < 0.005) (Fig. 6a). We then analyzed Aβ_42_ plaques in the brain (Fig. 6b). Although the average size of the Aβ_42_ plaques in both TP1 and TP2 brains became smaller than that in the sham brains (*p* < 0.01), the brains from TP2 mice contained a significantly lower number of plaques than them from the sham mice. These results indicate that the transplanted hpMSCs can suppress activation of the resident immune cells and formation of toxic plaques over time. Moreover, the results show that double treatment may more efficiently inhibit the progression of AD-like brain degeneration.

## Discussion

In this study, we investigated the feasibility of stem cell therapy for TBI using hpMSCs and different time windows for TBI treatment with a controlled cortical injury (CCI) model. Compared to other widely used TBI animal models such as fluid percussion injury (FPI) and weight drop models, the CCI model is more reproducible and applicable to various species, including mice, rats, ferrets, and swine, because it is generated by an electromagnetic impact device. The CCI model also represents widespread damage in humans, including acute cortical loss, axonal injury, damage to the blood-brain barrier, hippocampal and thalamic injury, and experiences of cognitive impairments. Because the CCI model allows for a biochemical investigation, which is not available in an FPI model, and the information from the CCI model is applicable in other species, the hpMSCs were infused into the CCI model to explore both pathological and therapeutic mechanisms [2, 27]. The main observation here was that hpMSC treatment at two time points (TP2), 4 and 24 h post-injury, could more efficiently reduce infarct size and enhance the recovery of sensorimotor functions compared to sham and single treatment at 24 h post-injury (TP1) strategies, which is a widely employed procedure [28–30]. Our results suggested that the more favorable outcomes from double transplantation resulted from the modulation of the early responses after damage, such as suppression of oxidative stress and inflammatory responses, activation of astrocytes/microglia activation, and enhancement of angiogenesis and TNFR survival signals.

According to *the Guidelines for Surgical Management of TBI*, the recommended time windows for surgery and treatment are variable depending on the types of injuries [12, 31, 32]. In epidural hematoma and subdural hematoma patients, surgical treatment within 2 h after injury enhanced recovery compared to a later operation [33]. Another group found that treatment for more than 6 h after injury failed to produce beneficial effects [34]. For traumatic parenchymal lesions, Jane’s group reported that an operation at less than 48 h after injury increased the probability of a promising outcome compared to surgery after 72 h post-injury [35]. Another group conducted operations in 27 children who suffered from injury and found that early treatment at a median of 19.2 h after injury showed a promising trend toward augmented recovery, which was not statistically significant [36]. These equivocal results may come from the heterogeneous nature of TBI in which multiple cues lead to multi-directional disturbances. The spectra of efficacies encourage us to develop new therapies that can modulate multiple signals systemically in different types of TBI. Recently, remarkable advances have been achieved in stem cell therapy for acute brain damage because stem cells are able to modulate the microenvironment and can invigorate endogenous survival signals and regeneration [37]. However, information on the appropriate treatment and mechanism is still scarce in both preclinical and clinical fields. In many animal models, the transplantation time points ranged from 2 h to 2 months post-injury and the number of injected cells was different from experiment to experiment [38].

To solve the ambiguousness of the treatment, we looked into the response patterns of cells against damage in the in vitro scratching model of TBI. We found that the damaged cells displayed temporal responses in inflammatory genes at two time points with a different pattern: expression of all three genes, IL-1β, TNF-α, and IFN-γ, increased within the first 4 h in the scratched HT22 cells as immediate early responses, but expression of IFN-γ and TNF-α declined, followed by rebounding after 12 h after stress. However, IL-1β expression resumed an increase in the cells at 12 h after damage, coming after maintenance until 8 h post-injury. The recurrence of enhanced expression at later time points represents late responses in the damaged cells (Fig. 1a). These temporal inductions may be ascribed to the NFκB-associated modulation because all three genes are known to be regulated by NFκB, whose transcriptional activity increased within 24 h following scratches and other stressors, such as irradiation and lipopolysasccharide (LPS) [39–42]. Although the time course of NFκB activity in scratched cells has not yet been studied, NFκB activation in the immediate early phase of irradiation injury triggered induction of cytokine, such as IL-1 and TNF-α, and the concentration of NFκB p65 in human skin fibroblasts increased immediately after 50 cGy irradiation, followed by a reduction and second increase before 10 h post-irradiation as the second surge in the scratched HT22 cells [39, 43]. Given that NFκB activity is modulated by IκB regulatory signals and other collaborating transcription factors, such as nuclear factor of activated T cells (NFAT), whose activities are regulated by diverse signals, including ROS, cytokines, growth factors, metabolic status, and injury [44], the temporal responses are associated with multiple factors that regulate NFκB signaling in the damaged HT22 cells. Another possible signal may be mediated by the early activation of AKT survival signals: the damaged cells activate an AKT survival signal for up to 4 h and are resistant against cell death in both mouse and rat TBI models. The AKT activation then declines by 24 h [45, 46]. The presence and maintenance of the survival signals, including the AKT pathway, may be critical for rapid recovery in TBI patients. This idea is partly reinforced by our in vivo results. Compared to sham mice, TP2 mice exhibited a significant induction of the activated AKT level. With a trend in elevation of anti-apoptotic Bcl-xL, these upregulated survival signals have roles in the mitigation of brain injury at early time points (Fig. 4). Consistent with these enhanced early survival signals, a smaller infarct size at 3 and 7 days after injury was detected in TP2 mice compared to sham and TP1 mice (Fig. 1). While we prepared the manuscript, Peng et al. reported a meta-analysis of the efficacy of MSCs in TBI animal models [38]. After analyzing 24 publications that contained well-controlled systems among 924 journals, they found that MSCs, especially umbilical cord blood MSCs, are capable of recovering sensorimotor and neurological functions and that an intravenous injection of 1-6 × 10^6^ cells into male SD rats at 6 h post-injury showed the greatest benefit among the experimental systems. Although a variety of damage types, species, and injected cells were analyzed, only four mouse TBI systems transplanted at 24 h post-injury were evaluated. When more information on the mouse system is completely necessary, our results now present valuable evidence for the development of appropriate TBI treatment.

IL-6 is reported to function as a pro- and anti-inflammatory factor and as an angiogenic factor via VEGF-α induction [47]. Figure 2c shows concomitant induction of IL-6 and VEGF-α in TP2 mice, indicating that the double-injected hpMSCs can modulate IL-6- and VEGF-α-mediated angiogenesis. However, another angiogenic factor regulated by IL-6, Nrp-1, was significantly suppressed in TP2 mice. Because Nrp-1 can also modulate regulatory T cell-mediated antigen recognition [48, 49], VEGF-α and Nrp-1 in our system may be differentially regulated by IL-6 and the regulation of Nrp-1 by hpMSCs plays a role in hpMSC-mediated immune modulation. This is an intriguing quandary to be further studied.

We looked into the underlying mechanism of hpMSC-mediated TBI recovery and found that the double injection-induced expression of TNF-α and FasL was accompanied by the reduced expression of caspase 8 at the early time point. TNF-α and FasL are known to activate necroptosis through the death-inducing signaling complex (DISC) and activation of caspase 8. Conversely, they also induce survival signaling through the formation of complexes with TNFR2, TRADD, TRAF2, RIP1, and cIAP and activation of NFκB-associated survival signals [50]. In our system, TP2 mice exhibited inductions of TNFR2, TRAF2, cIAP2, and p50 NFκB expression compared to sham and TP1 mice (Fig. 3), indicating that the double transplantation-mediated efficient recovery is partially due to the activation of TNFR/Fas survival signals, which has not yet been reported. The role of TNFR/Fas-mediated cell death in TBI has been reported: TNF-α and Fas are induced following TBI and TNF-α/Fas double knockout mice exhibit amended motor and spatial memory functions with less brain damage in TBI models [51, 52]. Lotocki et al. also reported alterations in the TNFR-mediated signals in their TBI rat model. Under normal conditions, a portion of TNFR forms the survival complex in lipid rafts, but upon brain damage, TNFRs, not Fas, were redistributed on the membrane and activated caspase-8-mediated apoptosis [53]. Whether stem cell treatment can modulate the TNFR distribution and activate their survival signals in various injuries, including TBI, remains unknown. Interestingly, TP1 mice also expressed some components of the TNFR survival signals, but their restoration capacities were limited compared to TP2 mice, suggesting that TNFR survival signals are necessary but not sufficient for efficient rehabilitation. Upon the differential modulation of inflammatory responses in TP1 and TP2 mice, the combination of TNFR signal activation and regulation of immune responses may lead to more efficacious outcomes in TBI treatment. This hypothesis warrants further investigation.

The double injection at 4 and 24 h after injury showed advantages in the enhancement of anti-inflammatory and pro-survival signals compared with a single injection at 24 h post-injury. Considering the kinetics of various cytokines in CCI TBI animal models and TBI patients and also in our in vitro results, the increased restoration by hpMSC infusions at 4 and 24 h after injury is reasonable [20, 54–56]. Many early responding cytokines, including IL1-α, IL1-β, IL17, RANTES, and TNF- α, reached their peaks in gene expression or protein in the blood within 6–12 h after injury, and then their expression declined within 24 h post-injury. In particular, MCP-1 (CCL-2), which is a key chemoattractant protein in macrophage recruitment into damaged areas, was maximally expressed within 6 h and then maintained in CCI mice. In the closed TBI model (CHI), the MCP-1 protein level in the serum was enhanced at 4 and 12 h after injury, followed by a reduction. Although we cannot exclude the possibility that injections at later time points produce better outcomes, upon the kinetics of the key cytokines, we argued that the double injection at early time points, 4 and 24 h post-injury, could be more effective in the recovery of injury-mediated deficits in animals compared to a single injection at 24 h after injury. In our system, we cannot rule out another possible explanation that a single injection of hpMSCs at 4 h post-injury could make the difference, and the additional infusion at 24 post-injury had a minimal contribution. Although many cytokines reached their peaks and were subdued within 24 h, the key phagocytic cell-recruiting chemokines, CCLs, were maintained for more than 25 h [20]. This prolonged activation of subset cytokines may explain the maximum macrophage infiltration at 66 and 72 h post-injury [57]. Consistent with these results, a meta-analysis of the TBI models suggested that MSC injections at 6 h post-injury showed better efficiency compared to 2, 3, and 24 h after insults [38]. However, their analysis showed that any administration before and after 24 h post-injection generated huge variations and differences. Due to the multiple signal pathways and mechanisms in TBI pathogenesis, a single injection at an early time point could work, but may not guarantee favorable outcomes in the recovery of the complicated deficits as much as the additional injection at a later time point.

In addition to the efficient modulation of inflammatory and survival signaling, hpMSC transplantation was able to suppress the formation of Aβ_42_ plaques in TBI mice at 10 weeks post-injury. We already reported that hpMSC-transplanted AD mice displayed reduced Aβ plaques and enhanced spatial memory functions [16]. Although the working mechanism is under investigation, in our system, double-injected mice exhibited reduced formation of neurotoxic Aβ_42_ plaques in the TBI brain. Due to the intravenous injection, the hpMSC-mediated influence is systemic and paracrine. To identify the pertinent factors, we analyzed metabolites and compounds using conditional media after hpMSC culture. This study will allow for the identification of new drugs that inhibit the mechanisms of AD and TBI.
